# Accuracy of Robotic-Guided Pedicular Screw Insertion in Thoracolumbar Spinal Surgery

**DOI:** 10.3390/jcm14238425

**Published:** 2025-11-27

**Authors:** Ignacio Dominguez, Rafael Luque, Angela Carrascosa, Juan Pablo Castaño, Alicia Collado, Pedro Alonso Lera, Fernando Marco

**Affiliations:** 1 Department of Orthopaedics, Hospital Clínico San Carlos, 28040 Madrid, Spain; ndominguez03@gmail.com (I.D.); rafaluqueperez@gmail.com (R.L.); acollado@gmail.com (A.C.); 2 Department of Neurosurgery, Hospital Clínico San Carlos, 28040 Madrid, Spain; amaca3582@gmail.com (A.C.); dr.juanpablocm@gmail.com (J.P.C.); palolera@hotmail.com (P.A.L.)

**Keywords:** thoracolumbar spine, spinal disorders, robotic surgical procedures, pedicle screws, spinal fusion

## Abstract

**Background/Objectives**: Screw placement accuracy with robotic guidance shows better results than freehand techniques, yet there is scant data for region-specific outcomes, fusion rates, and complication profiles in different populations. The purpose of this study is to assess and describe screw-rod accuracy, fusion rates, and complications of robot-assisted pedicle screw placement in a Spanish cohort. **Methods**: Robotic-guided technique for thoracolumbar fusion in 115 patients (July 2020–January 2025) using ExcelsiusGPS^®^ platform (Globus Medical Inc., Audubon, PA, USA). Inclusion criteria: adults (≥18 years) with postoperative CT to assess accuracy. Primary outcomes were screw accuracy (assessed on the Gertzbein–Robbins scale), fusion rates, and complications (infection, osteolysis, and subsidence). Jamovi 2.6 was employed for statistical analysis. **Results**: Of a total of 726 screws implanted, 590 screws (95 patients) were studied: 584 (99%) Grade A, 5 (0.8%) Grade B, and 1 (0.2%) Grade C. Fusion was achieved in 85 out of 95 patients (89.5%). Complications included superficial infections (3/115 patients, 2.6%) and asymptomatic osteolysis (8/95 patients, 8.4%, mostly at S1). There was no screw subsidence or removal. **Conclusions**: Robotic-guided pedicle screw placement demonstrated exceptional accuracy (99% Grade A), high fusion success (89.5%), and minimal complications in this Spanish cohort, supporting its clinical utility for spinal instrumentation.

## 1. Introduction

Spinal disorders that require surgery are becoming more widely acknowledged as a global health concern, mainly due to population aging and the increase in degenerative illnesses [1]. Over the past 15 years, spinal procedures have increased by 2.4 times [2]. Minimally invasive (MIS) techniques have been included in spinal surgery in many ways, including percutaneous pedicle screws fixation. Since screws are the main anchors in posterior spinal fusion procedures, it is essential to insert them precisely. Despite the relatively few clinical adverse events linked to malposition, misplaced screws can lead to dural tears [3], neurological/neurovascular injuries [4], sub-optimal biomechanics [5], and other visceral involvement [6]. Additionally, it is believed that up to 15.7% of cases with pedicle malposition go unreported [3]. In the meantime, the prevalence of pedicle screw problems varies greatly, from 1% to 54%, emphasizing the significance of anatomical features and patient-specific factors, including osteoporosis and deformities, in achieving successful screw insertion [7]. MIS modalities mainly include computed tomography navigation (CTnav), fluoroscopic guidance, and robot-guided surgery. Multiple systematic reviews have reported substantially growing evidence in all three fields [8,9]. While fluoroscopy alone has been reported to have a 91.3% accuracy [8], it has some drawbacks, like the obvious radiation exposure risk and ease-of-use limitations [10], as well as the level of surgeons’ experience, which introduces a degree of unpredictability. On the other hand, both CTnav and robotic-guided surgery have been reported to have higher predictability irrespective of the surgeons’ level of experience (7.0% [11] and 5.1% [12] vs. 15.7% [3], respectively) [3], and robotic-guided surgery is still reported to have higher accuracy compared to fluoroscopic guidance [11]. Furthermore, several recent meta-analyses and reviews have confirmed that robotic-assisted techniques achieve a far greater percentage of “perfect” (Grade A) and “clinically acceptable” (Grade A + B) pedicle screw insertions than freehand or fluoroscopic-guided methods. This advantage in radiographic accuracy is now well-established [13]. Less postoperative pain, faster recovery times, and less muscle damage have made robotic guidance more widely used [14]; however, this selection’s main drivers are accuracy, safety, and cost. Despite these advantages, significant barriers and confounding variables remain. The substantial capital investment and per-procedure costs are juxtaposed against an emerging body of literature arguing for its long-term cost-effectiveness by reducing revision rates and hospital stays [15,16]. Furthermore, the steep and highly variable learning curve, which one recent meta-analysis suggests may require 60 cases for many surgeons to overcome, acts as a significant clinical confounder [17]. Similarly, patient-specific factors, particularly poor bone quality, present a crucial challenge; a 2024 systematic review identified osteoporosis as a significant risk factor for mechanical complications like screw loosening and pseudarthrosis, which can mask the benefits of precise, robot-guided instrumentation [18]. These factors, along with case-specific challenges like high BMI or instrumentation in the technically demanding thoracic spine, complicate the interpretation of clinical outcome data [19].

These difficulties have created a crucial research gap. Numerous meta-analyses have demonstrated radiographic accuracy of robotic guiding, but there is still much disagreement about whether this translates into clearly better clinical outcomes. For example, recent systematic reviews and large-scale studies have found that patient-reported outcomes, complication rates, and revision rates do not significantly improve because of this precision [20]. Other recent meta-analyses, on the other hand, directly oppose this, stating that robotic aid does lead to decreased rates of surgical revision and postoperative complications [21,22]. This “accuracy-clinical outcome paradox” implies that the advantages of robotics might be strongly influenced by technology, surgical procedure, patient group, and handling of variables such as bone health and learning curve.

To explain this disparity, the field urgently needs high-quality and region-specific data. This study used robotic guidance to evaluate pedicle screw insertion accuracy, fusion rates, and complications in the Spanish population.

## 2. Materials and Methods

### 2.1. Study Design and Ethics

This is a consecutive, multicenter, observational, single-arm retrospective analysis study. During July 2020 to January 2025, adult patients who had robotically assisted pedicle screw implantation were included in this study. Due to its retrospective nature, the study was conducted in accordance with institutional ethical standards and applicable regulations.

All the data was anonymized and managed in compliance with the institutional data protection policies.

### 2.2. Patient Selection

Inclusion criteria were as follows:

- Age ≥ 18 years.
- Undergoing spinal fusion surgery involving thoracic, lumbar, or sacral pedicle screw placement.
- Use of robotic guidance with the ExcelsiusGPS platform (Globus Medical Inc., Audubon, PA, USA) during screw placement.
- Availability of postoperative CT imaging for accurate assessment.

Exclusion Criteria were as follows:

- Patients younger than 18 years.
- Procedures not involving pedicle screw instrumentation.
- Cases in which robotic guidance was not utilized or was converted to freehand or navigation-assisted techniques intraoperatively.
- Lack of postoperative CT imaging resulted in exclusion from the screw accuracy analysis only, but such patients were retained for all other clinical and radiological outcome analyses.
- Incomplete or missing clinical or radiological data relevant to the analysis.
- Revision surgeries.

Data collected included age, sex, clinical diagnosis, and presence of deformity, operative data including the treated anatomical levels, invasiveness, type of implants, preoperative/postoperative CT scans, fusion rates, and complications. Complications included infection, intervertebral cage subsidence, and osteolysis.

### 2.3. Robotic Workflows and Surgical Technique

The Excelsius GPS (Globus Medical Inc., Audubon, PA, USA) platform was used for all surgeries. Depending on the surgical needs, two workflows were used.

**Figure 1:**
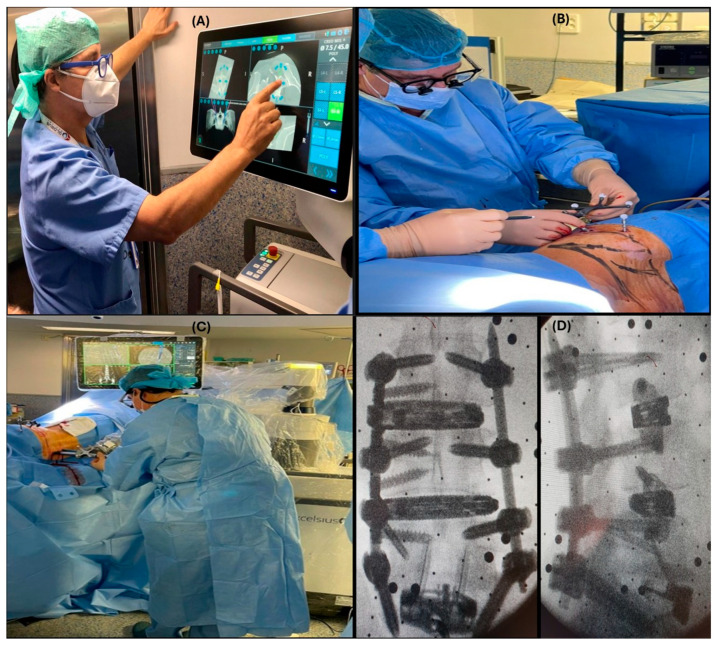
Preoperative workflow: (**A**) Scan and plan, (**B**) single lateral position DRB and SM in the left iliac crest, (**C**) percutaneous pedicular screws in lateral position, (**D**) intraoperative X-rays. Single-position surgery. L3-S1 percutaneous pedicular screws. ALIF, OLIF, and XLIF.

Preoperative workflow: A preoperative CT (Figure 1A) of the affected area is loaded by robotic computers. At this point, the surgeon plans the position of the implants and cages as well as trajectories and alignment. Once the patient is positioned on the operating table (prone or lateral) and after draping the surgical field, DRB (Dynamic Reference Base) and SM (Surveillance Marker) were positioned in both iliac crests (Figure 1B,C). A 2D X-ray is taken using a conventional C-arm. These images are merged with the preoperative ones (Figure 1D). As a result of this procedure, a virtual 3D image is available to start surgery.

Intraoperative workflow: In those cases, in which it is necessary to do any bone work that eventually changes anatomy, or when we are operating on specific anatomical areas such as the upper thoracic or cervical spine, it is mandatory to acquire intraoperative 3D images. In this study and all cases, this was performed using the Ziehmn Vision RFD 3D system (Ziehm Imaging, Nuremberg, Germany). After positioning, the DRB and SM were placed on the left iliac crest. An ICT is placed over the area to be intervened, and a 3D scan is performed. The surgeon plans the position and trajectories of implants, and the procedure is ready to start.

A final real image is taken before the patient leaves the operating room to confirm the correct position of the implants.

### 2.4. Screw Accuracy and Fusion Assessment

**Figure 2:**
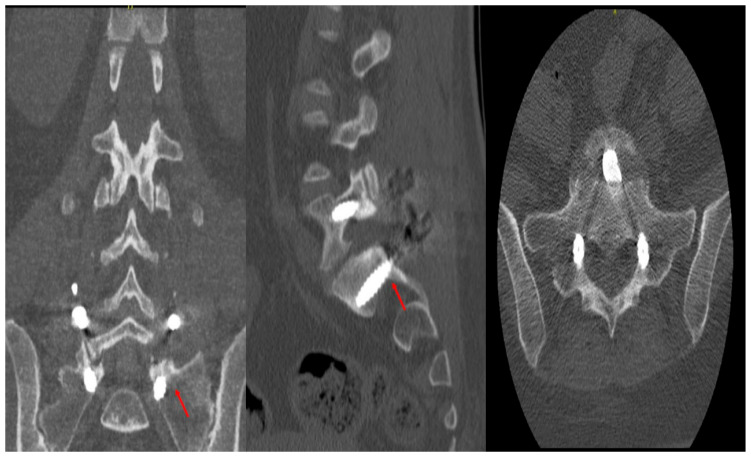
A four-grade system (A to E) was created to determine the pedicular screws’ position in a postoperative CT scan of the instrumented area. In this images, it is possible to see a Grade B medial breach antero-posterior, lateral, and axial views in the CT scan.

Accuracy of the pedicle screws was assessed using the classification of Gertzbein and Robins (CITA). A four-grade system (A to E) was created to determine the pedicular screws’ position in a postoperative CT scan of the instrumented area (Figure 2). Two fellowship-trained spinal surgeons who did not participate in the surgical procedures and were blind to all patient data and intraoperative results independently assessed postoperative CT scans. Axial and sagittal CT scans were used to assess screw accuracy using the Gertzbein–Robbins classification method. Consensus discussion was used to settle disagreements among reviewers to ensure consistent interpretation across all cases. Fusion was evaluated using postoperative CT scans obtained at approximately 6 months, defined using a bone trabecula passing through the affected area, consistent with our institutional follow-up protocol. We conducted a patient-level analysis, meaning that fusion status was assessed per patient rather than per individual spinal level.

### 2.5. Statistical Analysis

All statistical analysis in this study was performed using Jamovi software (Version 2.6). Categorical variables were presented as numbers and percentages, while continuous outcomes were reported as mean ± standard deviation if normally distributed and median (IQR) if the data were skewed. The normality of data was tested using the Shapiro–Wilk test. No inferential or regression analyses were performed, as the dataset lacked a control group and sufficient variability to support meaningful comparative or predictive modeling.

## 3. Results

### 3.1. Patient Population

In all, 115 patients were involved in this study. The median age was 64 years (IQR: 54, 64), with 52 (45.2%) being male. Most of the patients were diagnosed with degenerative discopathy (DDD), 56 (47.8%), or spondylolisthesis, 39 (33.9%). Seventeen patients (14.8%) had a deformity at diagnosis. See Table 1 for more data.

### 3.2. Surgical Data

In total, 726 screws were implanted, with 90 patients having interbody implants. The Altera banana expansion intervertebral cage was the most used, 48 (41.7%). All patients were operated on via a posterior approach, while 18 (15.7%) patients were operated on using an anterior approach simultaneously. A total of 89 (77.4%) underwent minimally invasive surgery (MIS), and 26 (22.6%) underwent open surgery. Table 2 summarizes the surgical data.

### 3.3. Screw Accuracy and Fusion Rate

Graded according to Gertzbein and Robbins system (GRS), pedicle screw accuracy and fusion rates were available for 95 (95/115) patients implanted with 590 (590/72631) screws. Overall, there were 584 (99%) screw Graded A and 5 (0.8%) graded B, only 1 (0.2%) graded C, and there were no screws graded D or E. GRS grades by level, diagnosis, type of anterior approach, type of posterior approach, and implant type are presented in Table 3, Table 4 and Table 5.

Fusion has been achieved in 85 (85/95) patients. And 10 patients showed no fusion diagnosis yet, types of posterior approach, anterior approach, and Implant are shown in Table 6.

### 3.4. Complications Resulting from the Procedures

Infection was reported in three (2.6%) patients. All of them were superficial, occurred in open procedures for Adult deformity, and were solved completely after debridement, lavage, and specific antibiotic therapy during four or six weeks, depending on the microbiologist’s choice. There was no screw revision or intervertebral cage subsidence. Osteolysis occurred in eight (8.4%) patients; most of them (five screws) were at the S1 level. All of them were asymptomatic. Those in S1 appeared in long fusions (above L2) with a TLIF technique, in which it was not feasible an ALIF due to vascular anatomy. No screws were removed from patients (Table 7).

## 4. Discussion

The primary goal of emerging robotic spinal surgeries is to enhance the accuracy of screw placement compared to conventional freehand techniques, as screw misplacement can lead to significant complications, including revision surgeries and neurological or vascular deficits [23]. Therefore, robotic surgeries aim to decrease postoperative complications and intraoperative errors, helping reduce revision surgeries and follow-up visits [24]. However, to create a measurement tool for screw placement accuracy, Gertzbein and Robbins developed a scale that starts with Grade A for optimal positioning with no cortical breach [25]. Grade B and C correspond to cortical breaches smaller and larger than 2 mm, respectively. Grade D indicates cortical breaches between 4 and 6 mm, while Grade E is for breaches larger than 6 mm.

On this scale, our study of 726 screws and 115 patients found that 584 (99%) screws were graded A, 5 screws (0.8%) were graded B, and 1 (0.2%) was graded C.

Our remarkable accuracy and fusion rates are validated by recent systematic reviews. For instance, a 2024 meta-analysis of 21 TLIF trials [26] revealed that robot-assisted surgery produced 74% fewer proximal facet joint violations and about 12% greater “perfect” (Grade A) screw accuracy than fluoroscopic freehand (RR≈1.12). Overall, surgical durations were similar to traditional methods, even though robotic cases in that analysis had lengthier setup and operating times in RCTs. As most series claim ≥95% Grade A placement, our 99% Grade A rate is in line with the top outcomes in the current literature. Our fusion rate (89.5%) is also consistent with recent publications; Chang et al., for example, reported approximately 87% interbody fusion at 2-year follow-up in a robot-assisted TLIF group [27]. These comparisons verify that our accuracy and fusion results are in line with existing standards.

Our findings also align with Khan et al., who studied 75 screws and 20 patients who underwent robotic screw placement for degenerative spinal pathologies, found that 74 (98.7%) of their screws were graded A, while only one screw was grade B [28]. Additionally, in a prospective analysis, Lonjon et al. found similar accuracy in a cohort of patients with degenerative spinal diseases who underwent robotic spinal surgeries, with 97.3% of screws placed at a grade A and B [29]. Similar results were also observed in a meta-analytical study of 6041 pedicle screw placements found that the robotic spinal surgery group doubled the rates of grade A optimal positioning compared to the freehand group, with an odds ratio of 2.43 (*p* < 0.001) [30]. This accuracy, attributed to detailed multi-dimensional imaging and continuous optical tracking of screw trajectories of robotic surgeries, reduces not only bony breaches but also damage to adjacent neural structures, such as leakage of cerebrospinal fluids or injury of nerve roots, whereas freehand surgeries, which depend on the surgeon’s tactile feedback and anatomical landmarks, introduce considerable precision variability, especially in complex and challenging areas such as thoracic spines [29,31,32].

In contrast to our results, Ringel et al., in a randomized controlled trial of 60 patients and 298 screw placements, of which 146 were performed using a robotic system, found that 85% of screws were graded A and B [33]. However, this lower accuracy in their study resulted from improper fixation of the robotic arm and the movement of the cannula during the screw placement [34]. Similarly, Wang et al. studied 61 patients with degenerative spinal pathologies who underwent minimally invasive, robot-assisted TLIF surgery and found that only 85.4% (234 screws) were inserted without any cortical breach [34]. They attributed this reduced accuracy to the movement of guide pins or violations of the lateral vertebral wall during the pedicle screws placement. However, these differences in accuracy are often attributed to variations in robotic systems that have distinct accuracy rates, preoperative imaging, Kirschner-wires requirement, or techniques for mounting the robotic arm (to bone, table, or floor) [35]. In general, reduced screw placement accuracy in robotic assisted spinal surgeries could be explained by various mechanical factors, such as shifting, which is a change in the position of the robotic arm relative to the patient’s position, or skiving, in which vertical force on the surgical instrumentation, such as cannula or drills, deviate from the pre-established bonny trajectories [36].

Our study found that 432 screws out of 436 screws in TLIF surgeries were placed with no cortical breaches, while only four screws had cortical breaches of less than 2 mm. These misplaced screws were scattered among different patients; no patient had more than one breach, and none of the impacted instances showed clinical signs or needed additional care. These accuracy rates were higher than those of Zhang et al., who studied 100 screws and 50 patients who underwent robotic TLIF surgery. Among these 100 screws, 85 were graded A, while 13 and 2 screws were graded B and C, respectively [37]. On the other hand, all our PLIF screws achieved an optimal placement of Grade A. These results were also higher than those of Kim et al., who found that only 91% of robotic-assisted TLIF screws received Grade A, while 8% and 1% were graded B and C, respectively [38].

Effective radiographic monitoring of fusion success rates is crucial for determining patients who may benefit from subsequent or revision surgery. A successful fusion is achieved when bone growth extends across at least 50% of the intended fusion area while maintaining the bone density achieved immediately after surgery [39]. From a biomechanical perspective, radiographic confirmation of solid fusion across one side of the total fusion area is sufficient to indicate stable fusion, regardless of any radiolucent features on the contralateral side [34]. In this context, our study demonstrated a successful fusion rate of 89.5% (85 out of 95 patients), with half of the fusion failures (5 out of 10) occurring in patients with degenerative spinal pathologies. Similarly, Chang et al. reported an 87.3% interbody fusion rate among 26 patients with degenerative spondylolisthesis who underwent robotic-assisted TLIF surgery [27].

Additionally, three patients in our cohort had postoperative infections, and eight showed signs of osteolysis. Osteolysis is a biological reaction that occurs at the bone–implant interface, where implant particles stimulate macrophages, leading to osteoclast activation and bone resorption, which appears as radiolucency around the implant and results in progressive bone loss, periprosthetic fracture, and implant loosening [40].

When it is non-symptomatic, it needs careful attention. If osteolysis progresses or symptoms appear, revision surgery is mandatory.

In our cohort, osteolysis was detected radiographically as progressively lucent zones around the pedicle screws or implants, a recognized sign of implant-associated bone resorption [41]. All osteolysis cases were asymptomatic, so we managed them nonoperatively with close imaging follow-up. Only if radiographic lucency had progressed or symptoms of loosening appeared would we recommend revision surgery.

This approach follows standard practice, since isolated radiolucent “clear zones” (pseudarthrosis signs) are monitored unless clinical or imaging deterioration mandates intervention.

### 4.1. Learning Curve, Operative Time, and Economic Considerations

Robotic spine surgery is expensive and has a steep learning curve. Setup and operation times are more prolonged at the initial stage of the learning curve, but efficiency quickly increases with familiarity. For instance, Paramasivam et al. reported that following the first ~20 cases, robotic setup time decreased from 24 to 17 min [42]. Similarly, following the initial learning period, operational time gaps between robot and traditional processes tend to diminish. Economically speaking, robotic platforms are expensive to build and maintain (typically >USD500K–USD1.2M) [43]. There are conflicting published cost studies; some predict net savings from shorter OR times and lower incidence of complications, while others report higher index hospitalization costs because of longer surgeries and higher equipment costs [43]. When assessing robotic technology, these pragmatic aspects (learning curve and cost) must be taken into account in addition to the clinical advantages.

### 4.2. Strengths and Limitations

To the best of our knowledge, this is the first study to evaluate the efficacy and safety of robotic spinal surgery in a Spanish population, improving applicability in local clinical practice. Moreover, using the established Gertzbein–Robbins scale allows for a direct comparison between our results and those from other centers. Additionally, the inclusion of diverse spinal pathologies (degenerative conditions, spondylolisthesis, deformities, and pathological/traumatic fractures) alongside both minimally invasive and open surgical approaches strengthens the generalizability of our results across different clinical scenarios. Despite these strengths, the lack of a randomized controlled design limits our ability to attribute our results to robotic surgeries alone. Also, the retrospective nature of our study introduces potential selection bias that could limit our results in other settings. Moreover, the diversity of implant types and surgical approaches, while reflecting real-world evidence, introduces confounding variables that further limit our ability to isolate the direct impact of robotic surgery on our population. Another drawback is that only 95 out of 115 patients (82.6%) had postoperative CT imaging available, which may have introduced some selection bias into the accuracy analysis. However, we think this had little effect on the validity of the accuracy results because CT imaging was carried out in accordance with standard institutional procedures rather than intraoperative concerns. The heterogeneity of surgical approaches (open vs. MIS; anterior vs. posterior) and patient pathologies further confounds our findings. These factors limit causal inferences and underscore the need for future prospective, controlled studies. Regression or correlation analyses were not performed to determine predictors of fusion rates or screw inaccuracy. Such connections should be investigated in future prospective multi-arm studies with larger and more varied cohorts.

## 5. Conclusions

Although robotic-assisted spinal surgery shows encouraging screw placement accuracy and good fusion rates, our study’s retrospective, single-arm methodology suggests these results should be interpreted cautiously. Potential advantages of robotic assistance are supported by the capacity to precisely implant longer and biomechanically favorable pedicle screws, especially in MIS operations where anatomical landmarks are scarce. To ascertain if the enhanced technical results are translated into long-term clinical and financial benefits, more high-quality evidence, such as randomized controlled trials and cost-effectiveness studies, should direct wider implementation.

## Figures and Tables

**Table 1:** Demographics and baseline.

Variable	Data
Number of patients	115
Age (year), Median (Q1, Q3) (Range)	64 (54, 64) (16–91)
Sex	
Male, *n* (%)	52 (45.2)
Female, *n* (%)	63 (54.8)
Diagnosis, *n* (%)	
Degenerative discopathy	56 (47.8)
Spondylolisthesis	39 (33.9)
Adjacent Segment Degeneration	2 (1.7)
Adult deformity	11 (9.6)
Adolescent deformity	1 (0.9)
Pseudoarthrosis	1 (0.9)
Traumatic fracture	4 (3.5)
Pathological fracture	1 (0.9)
Deformity, *n* (%)	17 (14.8)
Levels treated, *n* of patients (%)	
T3	1 (0.9)
T4	3 (2.6)
T5	4 (3.5)
T6	3 (2.6)
T7	4 (3.5)
T8	3 (2.6)
T9	3 (2.6)
T10	5 (4.3)
T11	2 (8.7)
T12	10 (8.7)
L1	12 (10.4)
L2	17 (14.8)
L3	26 (22.6)
L4	83 (72.2)
L5	106 (92.2)
S1	71 (61.7)
Iliac	7 (6.19)

**Table 2:** Surgical data of the 90 patients.

Variable	*n* (%)
Total number of implanted screws	726
Invasiveness	
MIS	89 (77.4)
Open	26 (22.6)
Posterior approach types, *n* = 115	
TLIF	87 (75.7)
FTP	24 (20.9)
PLIF	4 (3.5)
Anterior approach types, *n* = 18	
ALIF	1 (0.9)
OLIF	1 (0.9)
XLIF	2 (1.7)
ALIF + OLIF	2 (1.7)
ALIF + OLIF (SP)	1 (0.9)
ALIF + OLIF + XLIF	6 (5.2)
ALIF + OLIF + XLIF (SP)	1 (0.9)
ALIF + XLIF	2 (1.7)
OLIF + XLIF	2 (1.7)
Types of Implants, *n* = 90	
Altera banana expansion cage	48 (41.7)
LRISE expansion cage	37 (32.2)
Static cage	5 (4.3)
PEEK	1 (0.9)
PEEK + Altera banana expansion cage	1 (0.9)
Total number of implanted screws	731
Invasiveness	
MIS	89 (77.4)
Open	26 (22.6)
Anterior approach types, *n* = 115	
TLIF	87 (75.7)
FTP	24 (20.9)
PLIF	4 (3.5)
Posterior approach types, *n* = 18	
ALIF	1 (0.9)
OLIF	1 (0.9)
XLIF	2 (1.7)
ALIF + OLIF	2 (1.7)
ALIF + OLIF (SP)	1 (0.9)
ALIF + OLIF + XLIF	6 (5.2)
ALIF + OLIF + XLIF (SP)	1 (0.9)
ALIF + XLIF	2 (1.7)
OLIF + XLIF	2 (1.7)
Types of Implants, *n* = 90	
Altera banana expansion cage	48 (41.7)
LRISE expansion cage	37 (32.2)
Static cage	5 (4.3)
PEEK	1 (0.9)
PEEK + Altera banana expansion cage	1 (0.9)

**Table 3:** GRS grading according to level.

Level Treated	Grade A	Grade B	Grade C	Grade D	Grade E
Overall, *n* = 590	584 (99%)	5 (0.8%)	1 (0.2%)	0 (0%)	0 (0%)
T4	4 (0.7%)	0 (0%)	0 (0%)	0 (0%)	0 (0%)
T5	4 (0.7%)	0 (0%)	0 (0%)	0 (0%)	0 (0%)
T6	2 (0.3%)	0 (0%)	0 (0%)	0 (0%)	0 (0%)
T7	3 (0.5%)	0 (0%)	0 (0%)	0 (0%)	0 (0%)
T8	3 (0.5%)	0 (0%)	0 (0%)	0 (0%)	0 (0%)
T9	2 (0.3%)	0 (0%)	0 (0%)	0 (0%)	0 (0%)
T10	3 (0.5%)	0 (0%)	0 (0%)	0 (0%)	0 (0%)
T11	14 (2.4%)	0 (0%)	0 (0%)	0 (0%)	0 (0%)
T12	15 (2.5%)	1 (0.2%)	0 (0%)	0 (0%)	0 (0%)
L1	21 (3.6%)	0 (0%)	0 (0%)	0 (0%)	0 (0%)
L2	24 (4.1%)	0 (0%)	1 (0.2%)	0 (0%)	0 (0%)
L3	45 (7.6%)	1 (0.2%)	0 (0%)	0 (0%)	0 (0%)
L4	139 (23.6%)	3 (0.5%)	0 (0%)	0 (0%)	0 (0%)
L5	137 (30%)	0 (0%)	0 (0%)	0 (0%)	0 (0%)
S1	120 (20.3%)	0 (0%)	0 (0%)	0 (0%)	0 (0%)
Iliac	8 (1.4%)	0 (0%)	0 (0%)	0 (0%)	0 (0%)

**Table 4:** GRS grading by diagnosis.

Diagnosis	Grade A	Grade B	Grade C	Grade D	Grade E
Degenerative discopathy	276 (46.9%)	4 (0.7%)	1 (0.2%)	0 (0%)	0 (0%)
Spondylolisthesis	160 (27.2)	1 (0.2%)	0 (0%)	0 (0%)	0 (0%)
Adjacent Segment Degeneration	5 (0.8%)	0 (0%)	0 (0%)	0 (0%)	0 (0%)
Pseudoarthrosis	10 (1.7%)	0 (0%)	0 (0%)	0 (0%)	0 (0%)
Adult deformity	118 (20%)	0 (0%)	0 (0%)	0 (0%)	0 (0%)
Traumatic fracture	8 (1.4%)	0 (0%)	0 (0%)	0 (0%)	0 (0%)
Pathological fracture	6 (1%)	0 (0%)	0 (0%)	0 (0%)	0 (0%)

**Table 5:** GRS grading by approach type and implant.

	Grade A	Grade B	Grade C	Grade D	Grade E
Posterior approach					
TLIF	432 (73.2%)	4 (0.7%)	0 (%)	0 (%)	0 (%)
FTP	120 (23.7%)	1 (0.2%)	1 (0.2%)	0 (%)	0 (%)
PLIF	12 (2%)	0 (0%)	0 (%)	0 (%)	0 (%)
Anterior Approach					
Not approached anteriorly	434 (73.6%)	4 (0.7%)	0 (0%)	0 (0%)	0 (0%)
OLIF	5 (0.8%)	0 (0%)	0 (0%)	0 (%)	0 (%)
XLIF	27 (4.6%)	0 (0%)	0 (0%)	0 (%)	0 (%)
ALIF + OLIF	18 (3.1%)	0 (0%)	0 (0%)	0 (%)	0 (%)
ALIF + OLIF (SP)	6 (1%)	0 (0%)	0 (0%)	0 (%)	0 (%)
ALIF + OLIF + XLIF	62 (10.5%)	1 (0.2%)	1 (0.2%)	0 (%)	0 (%)
ALIF + OLIF + XLIF (SP)	8 (1.4%)	0 (0%)	0 (0%)	0 (%)	0 (%)
OLIF + XLIF	24 (4.1%)	0 (0%)	0 (0%)	0 (%)	0 (%)
Implant type					
No implant	133 (22%)	1 (0.2%)	1 (0.2%)	0 (%)	0 (%)
Altera banana expansion cage					
	253 (42.9%)	3 (0.5%)	0 (0%)	0 (%)	0 (%)
LRISE expansion cage	128 (28.5%)	0 (0%)	0 (0%)	0 (%)	0 (%)
Static cage	21 (3.6%)	1 (0.2%)	0 (0%)	0 (%)	0 (%)
PEEK	6 (1%)		0 (0%)	0 (%)	0 (%)
PEEK + Altera banana expansion cage	6 (1%)	0 (0%)	0 (0%)	0 (%)	0 (%)
			0 (0%)		

**Table 6:** Fusion failure data.

Variable	
Number	10 (10/95)
Diagnosis	
Degenerative discopathy	5 (5.3%)
Spondylolisthesis	2 (2.1%)
Adult deformity	1 (1.1%)
Traumatic fracture	1 (1.1%)
Pathological fracture	1 (1.1%)
Posterior approach	
TLIF	4 (4.2%)
FTP	6 (6.3%)
Anterior approach	
Not approached anteriorly	9 (9.5%)
OLIF + XLIF	1 (1.1%)
Implant	
Not implanted	6 (6.3%)
Altera banana expansion cage	3 (3.2%)
LRISE expansion cage	1 (1.1%)

**Table 7:** Type of Complications.

Complication	*n* (%)
Infection	3 (2.6%)
Screw or cage subsidence	0 (0)
Osteolysis, Patients = 8 (8.4%)	
S1	5 (0.69%)
L1	2 (0.28)
L3	2 (0.28)
L4	1 (0.14)
L5	2 (0.22)
Screw removal	0 (0)

## Data Availability

All data relevant to the study are included in the article. Data may be available upon reasonable request from the corresponding author.

## Abbreviations

ALIF	Anterior Lumbar Interbody Fusion
DDD	Degenerative discopathy
DRB	Dynamic Reference Base
VR	Dynamic Reference (or Registration) Frame
FTP	Full Transpedicular
GRS	Gertzbein and Robbins system
ICT	Intraoperative Computed Tomography
LRISE	Lateral RISE Cage System
MIS	Minimally Invasive Surgery
OLIF	Oblique Lumbar Interbody Fusion
PEEK	Polyetheretherketone
PLIF	Posterior Lumbar Interbody Fusion
SM	Surface Marker
TLIF	Transforaminal Lumbar Interbody Fusion
XLIF	Extreme Lateral Interbody Fusion
ALIF + OLIF	Combined Anterior and Oblique Lumbar Interbody Fusion
ALIF + OLIF (SP)	Same-Patient Combined Anterior and Oblique Lumbar Interbody Fusion
ALIF + OLIF + XLIF	Combined Anterior, Oblique, and Lateral Interbody Fusion
ALIF + OLIF + XLIF (SP)	Same-Patient Combined Anterior, Oblique, and Lateral Interbody Fusion
OLIF + XLIF	Combined Oblique and Extreme Lateral Lumbar Interbody Fusion
IQR	Interquartile range
